# Medical Books

**Published:** 1847-04

**Authors:** 


ARTICLE X.

### MEDICAL BOOKS.

We noticed in our last number the enterprise of the Booksellers of Chicago, in bringing on large and well selected assortments of medical Books, and are happy to add that, as will be seen by reference to the cover, Messrs. Morrison & Talbott, of Indianapolis, are determined that the profession of their region shall be accommodated in like manner.

